# Toward Automating the Selection of Articles Reporting EQ-5D Data for Systematic Literature Reviews Using Large Language Models: Algorithm Development and Evaluation Study

**DOI:** 10.2196/86647

**Published:** 2026-08-31

**Authors:** Gábor Kertész, János Tibor Czere, Zsombor Zrubka, Laszlo Gulacsi, Marta Pentek

**Affiliations:** 1 Software Engineering Institute, John von Nemann Faculty of Informatics, Obuda University, Becsi str 96/B, Budapest, 1034, Hungary, +3616665528; 2 Laboratory of Parallel and Distributed Systems, Institute for Computer Science and Control, Budapest, Hungary; 3 Doctoral School of Innovation Management, Obuda University, Budapest, Hungary; 4 PSI CRO Hungary LLC, Budapest, Hungary; 5 HECON Health Economics Research Center, Obuda University, Budapest, Hungary

**Keywords:** BERT, BioBERT, large language model, systematic literature review, PubMed, health informatics, EQ-5D, quality of life, Bidirectional Encoder Representations from Transformers, Biomedical Bidirectional Encoder Representations from Transformers

## Abstract

**Background:**

Systematic literature reviews (SLRs) are essential for evidence synthesis in health research but remain labor-intensive, especially at the screening stage. Manual review of titles and abstracts requires substantial human effort, while existing automation tools still have limited adoption in health technology assessment. The EQ-5D questionnaire, a widely used patient-reported outcome measure for health-related quality of life, provides data that frequently underpin reimbursement and policy decisions.

**Objective:**

This pilot study evaluated whether recent large language models (LLMs) can support the identification of publications reporting EQ-5D data in PubMed records, using only publicly available metadata (title, abstract, and keywords).

**Methods:**

A total of 200 publications retrieved through the EuroQol PubMed filter were manually labeled by experts as reporting or not reporting EQ-5D data. The dataset was split into stratified training, validation, and test subsets. Several machine learning approaches were compared, including a Naïve Bayes baseline using bag-of-words features, a decision-tree model based on full-text keyword occurrence, and transformer-based LLMs (Bidirectional Encoder Representations from Transformers [BERT], Biomedical BERT [BioBERT], Scientific BERT [SciBERT], and Biomedical Language Understanding Evaluation BERT [BlueBERT]). Both classifier-only and fine-tuned configurations were tested across multiple learning rates. Model performance was assessed using accuracy, precision, recall, and *F*_1_-score.

**Results:**

Baseline approaches achieved near-random test performance (accuracy around 0.53). Classifier-only LLMs modestly improved results (accuracy up to 0.64 with SciBERT). Fine-tuned models substantially outperformed these baselines, with BERT and BioBERT achieving the best performance (accuracy=0.70; *F*_1_-score=0.68). In screening-oriented evaluation, this configuration achieved 90.0% sensitivity, 40.0% specificity, and 6 false negatives on the held-out test set. The models reproduced human screening tendencies despite the small dataset size, demonstrating the technical feasibility of LLM-assisted article selection.

**Conclusions:**

This study provides the first demonstration of LLM-assisted identification of EQ-5D data in biomedical literature. The findings support technical feasibility but do not establish a reliable stand-alone automated screening tool. Although limited by dataset size, the proposed workflow is reproducible and adaptable to other patient-reported outcome measures. Because validation was based on a single small train-validation-test split, the results should be interpreted as preliminary; future work will scale data collection, include statistical testing, and explore semisupervised learning to further reduce manual screening workload.

## Introduction

### Background

Systematic literature reviews (SLRs) are scientific methods to collate the available evidence, in order to answer specific research questions and support decisions. SLRs were first used in medicine to inform medical decision-making but currently are widely applied in various fields.

The methodology for conducting SLRs using electronic literature databases has been standardized to ensure reliable, high-quality, and reproducible findings and thus conclusions [1]. However, implementing the SLR method can be resource-intensive in terms of both time and qualified human effort [2]. Tasks such as constructing the search strategy, selecting relevant studies through multiple rounds with at least 2 independent reviewers, and extracting data require considerable resources. While the search is performed electronically using the search strategy designed for a specific research question and considering the features of the electronic literature database, the selection of records is a manual process. Two experts review the hits of the search independently from each other and assess which records fulfill the predefined eligibility criteria. This assessment is done first by the publication’s title, abstract, and keywords. For publications for which the decision regarding their eligibility was not possible in this round, undergo full-text review. In both phases discrepancies between the 2 independent reviewers are solved by discussions and a third reviewer can also be involved. This sophisticated selection method ensures, as far as possible, avoiding selection errors. However, the process becomes particularly challenging when the search yields a large number of results, such as several thousand.

These challenges have prompted the development of automation tools based on machine learning (ML) methods in the field of SLRs. The adoption of automation is still limited [3]; however, the literature on SLR automation is extensive and continually evolving [4]. Text classification methods have [5] improved since the breakthroughs of deep learning [6] and especially since recent advances with large language models (LLMs) [7,8].

Classifying text differs from general classification tasks, as text—represented as a sequence of characters—cannot be simply converted to a set of features. Early or classical approaches were based on character or word frequency, ignoring word order and sentences themselves. To improve performance on natural language understanding, researchers created models that represent semantic relationships in word sequences [9].

Initially, statistical tools formed the foundation of text analysis, with the bag-of-words (BoW) model being an example. This technique serves as a fundamental building block for various other tools. In this approach, the frequency of words in the text is counted, providing a representation based on their occurrence. While this method does not consider the specific positions of words in the text, it can be used as a feature extraction technique. Also, comparing word frequencies can indicate similarity between 2 texts; if the words and their frequencies match, the texts are considered similar [10].

A more effective solution involves representing words with their sequential order. Word embedding, or word vectorization, is an approach that represents documents and words as numeric vectors. This representation allows words with similar meanings to have similar vector representations and enables approximation of word meaning in a lower-dimensional space [11]. The input to this approach includes the word itself and its position within the text, which can be seen as a time series representation where words must follow each other in the same order.

However, it is worth mentioning that highly effective general models have only become available with the introduction of deep learning. Multiple methods for natural language processing (NLP) were developed, such as eLMo [12], Bidirectional Encoder Representations from Transformers (BERT) [13], and GPT [14]. eLMo, an NLP framework developed by AllenNLP, uses a 2-layer bidirectional language model (biLM) to calculate word vectors. The biLM consists of both forward and backward passes in each layer. eLMo generates embeddings for a word by considering the entire sentence in which the word appears. GPTs are a framework in the field of generative AI and fall under the category of LLMs. These models are artificial neural networks that use the transformer [15] architecture. Applications of GPT are well-known for their ability to generate human-like content.

BERT is a family of language models, introduced in 2018 by Google researchers. BERT is an unsupervised language representation model that deeply incorporates bidirectionality. It is pretrained on pure plain text corpora, considering the context surrounding each occurrence of a word. As a result, BERT generates contextualized embeddings that vary depending on the sentence. BERT is considered a more suitable approach for text classification compared to GPT, as the bidirectional processing of text allows the method to capture the context deeply; the latter is mostly used for generation.

The focus of our research is on the automation of SLRs in the field of health, specifically on a health outcome measure, the EQ-5D. Assessing health improvements from the perspective of patients has become a fundamental aspect of health care and the development of health technologies. Patient-reported outcome measures (PROMs) offer valuable insights into changes in health, functional status, and health-related quality of life (HRQoL) as perceived by patients [16]. PROMs are typically standardized, self-reported validated questionnaires that enable reliable and valid assessments of health outcomes.

The EQ-5D questionnaire is a PROM designed to evaluate and measure health. It was initially developed by the EuroQol Group in 1990 [17], and since then, numerous studies have used this measurement tool and its subsequent versions [18]. The advantage of EQ-5D among PROMs lies in its generic nature, allowing assessment and comparison of health status among patient populations with diverse diseases, as well as the general population. Additionally, EQ-5D data are commonly used to calculate health gains, expressed as quality-adjusted life years (QALYs), in health economic evaluations, thereby informing reimbursement and health policy decisions [19]. Consequently, there is a growing demand for SLRs that specifically focus on EQ-5D studies across various domains, with the aim of guiding clinical decision-making, developing public health strategies, conducting health economic evaluations, and performing health technology assessments. Despite the outstanding importance of access to EQ-5D data, to the best of our knowledge, no SLR automation tool specifically focusing on EQ-5D studies has been developed yet.

The aim of this pilot study was to evaluate the feasibility of automating the selection of publications reporting EQ-5D data based on publicly available metadata (title, abstract, and keywords) using LLMs. Rather than proposing a novel algorithm, the study focuses on the applicability of recent language model architectures to a domain-specific screening task in systematic reviews. Lessons learned from this small-scale experiment are intended to guide future methodological development and larger-scale validation studies.

**Figure 1.**
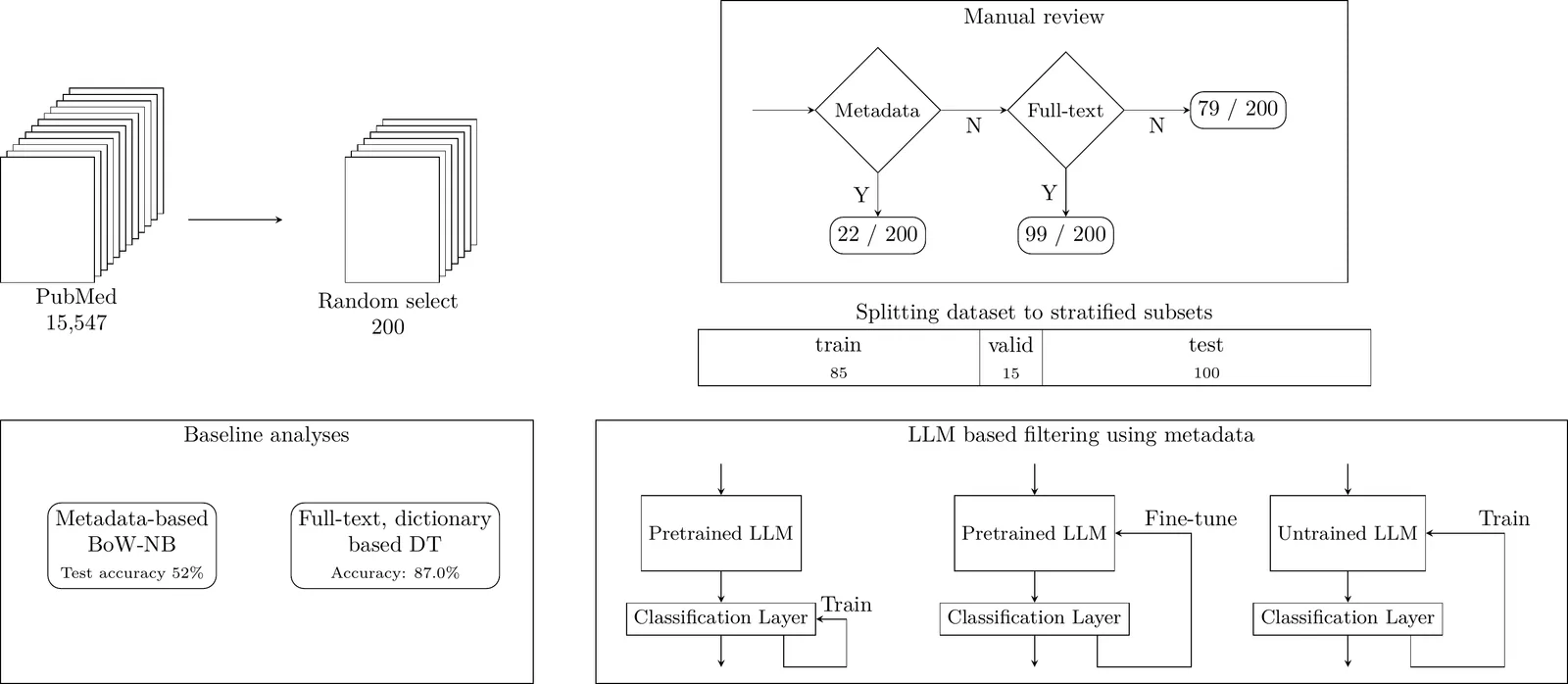
A brief overview of the methods applied during the research. BoW-NB: bag-of-words–Naive Bayes; DT: decision tree; LLM: large language model.

A large electronic database of biomedical literature, PubMed [20], was searched for EQ-5D studies. The curated and annotated dataset based on human selection work will be used as input for supervised learning, specifically a binary classification task, aimed at predicting whether EQ-5D data are reported in the full text of a given study by analyzing its title, abstract, and keywords. Different LLMs and training methods will be evaluated. Finally, lessons learned from this small experiment are discussed, and some points to consider in future research are formulated. An overview of the methods applied in the study is briefly summarized in Figure 1.

### Related Work

#### Transformer-Based Text Classification

Recent methods for text-based classification tasks are usually based on transformers [15], as these methods seem to outperform any other approach on widely used benchmark datasets [21-24]. For text classification, BERT is preferred over GPT architectures, as GPT models are autoregressive, while BERT is trained bidirectionally [25].

Applications of BERT for text classification tasks are used in many domains: from hate-speech detection in social media [26,27] to categorizing medical reports [28,29]. To apply a pretrained BERT model for a text classification task, there are a few approaches. Devlin et al [13] presented in the original paper describing BERT that fine-tuning is a straightforward method to specialize the pretrained model on a given task; source codes demonstrating these capabilities were also published. Lee and Hsiang [30] applied fine-tuning for patent classification, creating PatentBERT, which outperformed the state-of-the-art DeepPatent. Sun et al [31] presented that fine-tuning should be performed on a low learning rate to avoid catastrophic forgetting phenomena. Adhikari et al [32] applied the method to classify documents, outperforming other approaches on the benchmark datasets. Zheng and Yang [33] proposed a novel BERT-CNN method to improve classification performance.

Another approach for text classification is based on BERT-based feature extraction; in this case, the parameters in the pretrained model are untouched, and an additional layer or layers are added for classification. BERTScore [34] applied BERT to provide contextual embeddings to replace word embeddings, resulting in slightly different word representations based on context. Reimers and Gurevych [35] presented Sentence-BERT, which is a fine-tuned BERT model trained in a triplet network architecture for sentence embedding; the triplet loss function is based on the BERT feature vectors for the given anchor, positive, and negative pair.

#### Fine-Tuning of Language Models

While BERT itself is a model pretrained on general data [36], there are existing pretrained alternatives with scientific or biomedical domains, such as Scientific BERT (SciBERT) [37], Biomedical BERT (BioBERT) [38], or Biomedical Language Understanding Evaluation BERT (BlueBERT) [39]. It is also worth mentioning the Longformer [40], which is a transformer-based architecture overriding the typical token limitation of 512, resulting in high-performing models with 4096 or 16,384 sequence lengths. Other approaches on handling long sequences start with truncating; in many cases, simply truncating the sentence has only minimal effect on performance [41].

Regarding the training of BERT-based models, different methods were applied to improve performance. On fine-tuning, it is recommended to optimize the learning rate hyperparameter, as different settings have a significant impact on the resulting model [42]. It is worth mentioning that in multiple studies, the behavior of fine-tuning pretrained models is analyzed [43], and addressing instability [44] is a key question to achieve peak performance.

#### Screening Automation for SLRs

In the modern era of deep learning, and especially in the era of LLMs, multiple tools have emerged to support study screening for literature reviews. For instance, studies using active learning and classical text representations [45-47] (eg, Term Frequency-Inverse Document Frequency [TF-IDF] with logistic regression or Naïve Bayes) reported workload savings at 95% recall (WSS@95) in the range of 60%‐90%, although performance varies considerably across datasets and domains. GPT-based and related LLMs have recently been explored as tools to automate SLR screening without the need for task-specific training.

Studies have recently applied ChatGPT (OpenAI) for similar tasks [48,49]. A recent study by Guo et al [50] presented an automatic study screening method based on ChatGPT, with a problem very similar to that presented in this study. Results showed that the pretrained GPT-4 model as the backbone of the ChatGPT sessions provided high performance. Dennstädt et al [51] used 4 open-source LLMs (*Flan-T5, OpenHermes, Mixtrial,* and *Platypus2*) for title/abstract screening on biomedical reviews. They achieved promising sensitivity (82%‐98%) but at the cost of lower specificity (eg, 19%‐75%), indicating many false positives (FPs).

In parallel, the emergence of LLMs has enabled zero-shot and few-shot screening without task-specific fine-tuning [52,53]. Several recent studies have evaluated models such as GPT-3.5 and GPT-4 in systematic review workflows. Across both BERT-based and LLM-based approaches, a consistent pattern emerges: while transformer models can substantially improve screening efficiency and, in some cases, approximate human-level agreement, their performance is highly context-dependent and often unstable across datasets [51,54].

Overall, the SLR screening literature [55] provides strong evidence that ML can effectively support the process of study selection, particularly through prioritization and human-in-the-loop workflows. However, the task formulation in these studies differs from the presented work; instead of identifying studies that meet general inclusion criteria, they aim to predict reviewer decisions at the document level. In contrast, our setting requires predicting whether a publication contains extractable outcome data based solely on metadata, which represents a classification problem.

### EQ-5D Prediction

Regarding screening for EQ-5D, existing methods are designed to retrieve relevant studies [56,57] (eg, those reporting utility values or economic evaluations). Prediction or classification of individual records based on whether they contain extractable EQ-5D outcome data (eg, index values, visual analog scale (VAS) scores, baseline/follow-up measures, or group differences) instead of mentioning this measure is quite different.

To the best of our knowledge, there is no well-documented prior work that directly addresses this using automated methods; we did not identify studies that target the classification of publication metadata into those that contain EQ-5D outcomes and those that only reference the instrument.

## Methods

### EQ-5D Measurement Tool

The EQ-5D refers to a family of instrument versions of the original EQ-5D measurement tool. The original EQ-5D comprises 2 parts: a descriptive system and a VAS (EQ VAS) [58]. The descriptive system covers 5 health domains (mobility, self-care, usual activities, pain/discomfort, and anxiety/depression). The respondent is asked to indicate on a 3-level response scale the problem level that best describes his or her current health status (1: no problem, 2: moderate problem, and 3: extreme problem). Thus, 243 different health states (profiles) can be obtained by completing the descriptive system. Results can be presented as the proportion of the sample indicating different problem levels in each EQ-5D domain. An EQ-5D index score can also be linked to each health profile that reflects the utility (desirability and preference) the society attaches to each health state, where index score 1 represents perfect health, zero refers to the state of death, and negative values indicate health states that are considered as worse than death. The set of EQ-5D index scores (value set and tariffs) is typically country-specific, as it is obtained from the general population in a separate study (using direct utility measurements, mainly the time-trade-off method) [18,59]. The value set once established can be applied directly to calculate the EQ-5D index score from the responses obtained on the descriptive system.

The second part of the EQ-5D is the EQ VAS, a vertical 20 cm VAS, with end points of 0 and 100, representing the worst and 100 the best health, respectively, the respondent can imagine. Respondents are asked to mark on the EQ VAS how their health is on that day.

Since the development of the original EQ-5D, new versions have been validated and published, aiming to increase the sensitivity of the tool by applying 5-level response options (EQ-5D-5L version; hence, the original version was renamed as EQ-5D-3L), as well as to measure the HRQoL of children and adolescents with a “youth” version (EQ-5D-Y-3L and EQ-5D-Y-5L), which uses language adapted for children to describe health problems [60,61]. All these versions retained the original structure (descriptive system and EQ VAS). In parallel, different modes of administration (eg, digital and phone interviews) have been developed and the versions have been translated into more than 170 languages.

### Eligibility Criteria

Studies that report EQ-5D data from patients or the general population are considered eligible. Studies reporting results obtained on the EQ-5D descriptive system (ie, problem levels on the health dimensions) or reporting EQ-5D index scores are eligible, using either version (eg, EQ-5D-3L, EQ-5D-5L, and EQ-5D-Y) and administration method (eg, digital or paper-based or voice interactive system forms, self-complete or proxy version) of the instrument. Conceptual or methodological studies reporting the valuation, development, or psychometric properties of EQ-5D, or those that do not report index scores or descriptive results from actual patients or individuals, are considered out of scope.

Although EQ VAS is part of the EQ-5D instrument family, studies reporting only EQ VAS values were considered out of scope because the target of this screening task was the identification of studies reporting EQ-5D descriptive-system results or index scores suitable for health-state utility extraction and health economic evidence synthesis.

No restrictions are applied on the population in which EQ-5D was used; any age group, sex, condition, or geography, etc, is considered. Only full-text studies available in the English language are considered eligible.

### Data Source

PubMed [20] is a publicly available literature database containing over 36 million citations and abstracts of biomedical, life, and related sciences. PubMed is maintained and regularly updated with new publications and can be searched electronically. PubMed does not include the full text of the journals but generally provides links to relevant sources.

The EuroQoL Group developed a search filter to identify EQ-5D publications in PubMed. The filter is freely available on the EuroQoL website and can be combined with further search terms (eg, disease-specific terms). The exact search strategy is as follows:


(euroqol[All Fields] OR eq-5d[All Fields] OR eq5d[All Fields])

We conducted a search in PubMed using the EuroQoL search filter (without adding any further search terms) on October 13, 2022. The search resulted in a total of 15,547 records, published between 1990 and 2022. Altogether 200 publications were randomly selected from the results using the built-in sample command of Stata (version 2017; StataCorp LLC) statistical software. This set of 200 publications (published between 1999 and 2022) is used in the analyses.

### Data Preprocessing

#### Manual Review of the Set of Publications

All the 200 selected studies were collected in full text and examined by 2 independent reviewers based on the predefined eligibility criteria. Results were matched, and differences were discussed until a concealed position was reached. All publications were labeled indicating whether the publication includes EQ-5D data or not (true/false). All the resulting records consist of these values:

- Title
- Abstract
- Keywords
- Label (“true”/“false”).

It is important to note that while all PubMed exported records (metadata) are in English, the full text of the study may be in a different language. According to the predefined eligibility criteria, such studies were labeled as negative by the reviewers. This represents a practical limitation of metadata-based screening, since eligibility may depend on information not fully captured in the metadata.

#### Splitting the Data Into Training and Test Datasets

As the dataset is relatively small considering the varied contents and semantic diversity in scientific texts, 50% of the set is dedicated as the test subset, which was not directly involved in training; it was only used for performance measurement.

The remaining 100 records were split into a training and a validation subset with the lengths of 85 and 15, respectively. The training subset was used to tune and fine-tune the parameters of the model, while the validation subset was applied indirectly to forecast overfitting. The validation subset contains preselected elements, using a stratified splitting. Stratification was implemented using a fixed validation record ID set, resulting in the same ratio of positive and negative records as in the case of the training set.

As this is a pilot dataset, with a low number of samples, model performance estimates may be sensitive to the specific splitting strategy. Therefore, our setup is mainly used to provide an initial feasibility report.

### Baseline Analysis: BoW Model

Before applying advanced deep learning techniques, it is advised to check the performance of simple approaches as a baseline for our experiments. In the case of text classification, the previously mentioned BoW method is a straightforward selection.

This technique operates by systematically compiling the words within the text and counting their respective frequencies. Word occurrences define a feature vector for each record, which can be applied as an input for a simple classifier. Let’s define *V* vocabulary of words, with *w*_*j*_ representing the j^*th*^ word in *V*. Let *x_i_* ∈ *X* represent the input data, and *t_i_* ∈ *T* the input texts, where


$$x_{i}^{\left(j\right)}\text{=}\text{count}\left(w_{j},t_{i}\right),$$

for all,

A Naïve Bayes classifier based on the BoW methodology is trained and evaluated across identical subsets. The probability is based on the Bayes-theorem, having


$$P\left(c\text{|}x_{i}\right)\text{=}\frac{P\left(x_{i}\text{|}c\right)\cdot P\left(c\right)}{P\left(x_{i}\right)},$$

where P(a|b) represents the conditional probability of event *a* occurring if event *b* has occurred; and *c* defining the binary class of a negative or a positive sample, *c ∈ C*.

The Naïve Bayes classifier is a simple yet efficient method, which is based on a few assumptions regarding the data, features are independent and of equal importance. We applied a Gaussian Naïve Bayes classifier, where $P(x_{i}\text{|}c)$ is assumed to be following a Gaussian distribution. Based on the standard univariate Gaussian distribution,


$$P\left(x_{i}^{\left(j\right)}\text{|}c\right)\text{=}\frac{1}{\sqrt{2\pi \sigma_{c,j}^{2}}}exp\left(\text{-}\frac{{\left(x_{i}\text{-}\mu_{c,j}\right)}^{2}}{2\sigma_{c,j}^{2}}\right),$$

where *μ* represents the mean and *σ* represents the variance of the count of word *w*_*j*_ in class *c*.

When classifying, *P* (*x*_*i*_) can be ignored, as we assume that it is constant for all classes, *P* (*c*) can be given as


$$P(c)\text{=}\frac{\text{|}t\in T:\text{label}(t)\text{=}c\text{|}}{\text{|}T\text{|}},$$

which represents the ratio of class c elements among all data. To get the predicted class label $\hat{y}$ for a given feature vector *x*, P(c|x) has to be calculated for each class *c* as given in [eq:bow_classifier], and select the highest probability as the estimated label:


$$\hat{y}\text{=}arg\underset{c\in C}{max}P\left(c\text{|}x\right).$$

For the experiments, different metadata setups were checked: the title or the abstract itself, the title and the abstract together, and finally the title, abstract, and keywords concatenated together.

The BoW method starts with filtering and tokenization and removing stop words from text. The limit for the number of features was specified as 1000; however, this threshold was reached only when the subset including keywords was used. On limitation, features with small measured frequency are ignored.

### Baseline Analysis: Full-Text Analysis

To obtain information about EQ-5D data, the reviewers in most cases (89.0%) checked the full text. Although the aim of our study is to predict the availability of data based only on the metadata of a given publication, we have also prepared a method based on full text for comparison.

Full-text study analysis is a complex task. Based on the journals, the publishers, and even the scientific fields, the formats are widely different, with differences in markup language (HTML and TeX), format, or even plain text availability. As studies are usually available in a PDF, analyzing this format is more convenient.

The domain experts specified a list of keywords and phrases, mostly from the expressions often used in the EQ-5D descriptive system, which often indicates EQ-5D data in the study. The specified key phrases are index, value, utility/utilities, score, mobility, self-care, usual activities, pain, discomfort, anxiety, depression, looking after myself, doing usual activities, having pain or discomfort, feeling worried, feeling sad, and feeling unhappy.

To formally represent this method, let *k* ∈ *K* represent the abovementioned keywords, and function $C_{k}^{\left(d\right)}$ represent that document *d* contains keyword *k*, where $C_{k}^{\left(d\right)}:\{\text{true},\text{false}\}$.

A decision tree (DT) is a simple, data-driven ML method where the estimation of the trained model is explainable. A DT for binary classification can be represented as a function $f:\{0,1{\}}^{\text{|}K\text{|}}\to \{0,1\}$, where |K| is the number of input features, in this case, the number of keywords. Each element in the feature vector can only have 2 possible values: the text either contains the keyword or not; the output of the model is also binary.

Each internal node in the DT represents a decision based on a parameter; for this case, this can be formulated as


$$o_{j}\;\left(C_{k}^{\left(d\right)}\right)=\left\{ \begin{array}{ll} \text{true}, & \text{if }C_{k}^{\left(d\right)}\text{ is true},\\ \text{false}, & \text{otherwise}, \end{array}\right.$$

where *o*_*j*_ represents the decision of a given node in the DT, where the decision is based on keyword presence represented by $C_{k}^{\left(d\right)}$. The 2 outputs of the node represent the outcomes.

All leaf nodes in the DT define a class label, in this case either true or false. The estimated label is determined by the label associated with the leaf node reached by traversing the binary tree based on the decision rules.

During training, Gini impurity is applied during recursive partitioning to find subsets where most elements are with the same label. At each step the algorithm considers all possible splits and selects the set with optimal impurities; the process is continued recursively until further splitting does not improve the performance.

A PDF full-text processor was created in Python (Python Software Foundation), which checks every page of the provided input files for the given keywords or phrases until one is found in a given document; results are collected, and different keyword combinations are evaluated using DT.

### Selecting the Language Model, Pretraining, and Fine-Tuning

In order to select the best-performing pretrained LLM as the backbone of the filter, multiple domain-specific models were considered, detailed in Table 1. Where applicable, both cased and uncased versions were tested.

**Table 1.** The large language models applied in this study.

Name	Domain	Corpus	Sequence length
BERT^a^	General	BookCorpus, Wikipedia	512
SciBERT^b^	Scholar	Semantic Scholar	512
BioBERT^c^	Biomedical	PubMed	512
BlueBERT^d^	Biomedical	PubMed	512
SciBERT Longformer	Scholar	Untrained	4096

a BERT: Bidirectional Encoder Representations from Transformers.

b SciBERT: Scientific Bidirectional Encoder Representations from Transformers.

c BioBERT: Biomedical Bidirectional Encoder Representations from Transformers.

d BlueBERT: Biomedical Language Understanding Evaluation Bidirectional Encoder Representations from Transformers.

During the measurements, 2 major methods were applied to train the models:

- Applying the pretrained language model without altering the parameters, only training the classification layer
- Fine-tuning the pretrained model with a relatively small learning rate.

As the input consists of 3 separable texts, these were connected with the special token [SEP], resulting in a single input. In case the sequence length exceeds the maximal length defined by the models, truncation was applied at the sequence end.

#### Applying Pretrained Models

**Figure 2.**
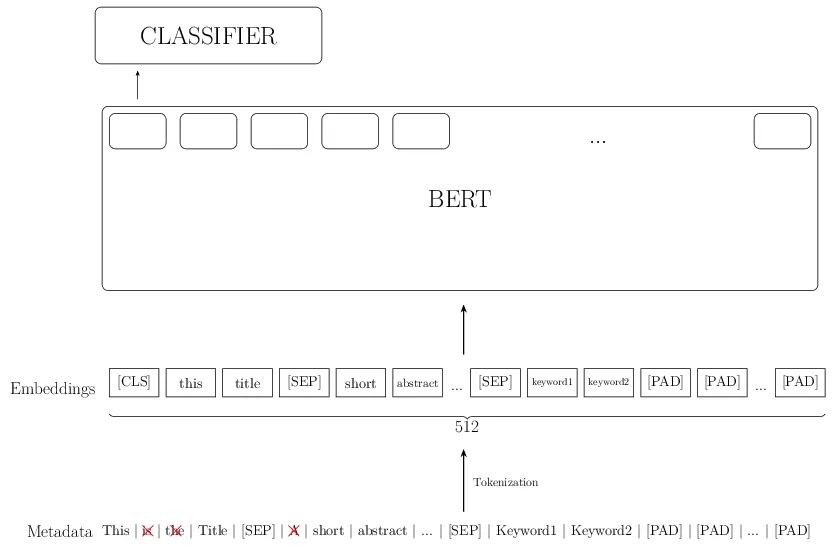
A brief summary of text classification using Bidirectional Encoder Representations from Transformers (BERT)–based large language models (LLMs). The paper metadata is concatenated, tokenized, and fed to the model as input, and finally a classification layer is built on the so-called pooled output of the network. On applying pretrained models, only the classification layer is trained; on fine-tuning, the parameters of the backbone BERT are also fitted. BERT: Bidirectional Encoder Representations from Transformers.

BERT-based models are prepared to be used for text classification tasks (Figure 2); a classifier layer is simply attachable to the pretrained model; during training this layer is tuned, and the model parameters remain unchanged.

To formally represent the applied method, let’s define BERT as the pretrained language model and $\theta_{\text{BERT}}$ as the parameters of the model. By having


$$\hat{y}\text{=}f_{\text{classifier}}\left(\text{BERT}\left(t,\theta_{\text{BERT}}\right);\theta_{\text{classifier}}\right),$$

where $f_{\text{classifier}}$ stands for the final classification layer and BERT(x;θ) represents the embeddings of *x* based on parameters θ. When training, only the parameters of $f_{\text{classifier}}$ are modified, represented as $\theta_{\text{classifier}}$; with having *L* as the loss-function


$$\underset{\theta_{\text{classifier}}}{min}\frac{1}{\text{|}T\text{|}}\sum_{i\text{=}1}^{\text{|}T\text{|}}L\left(f_{\text{classifier}}\left(\text{BERT}\left(t_{i};\theta_{\text{BERT}}\right);\theta_{\text{classifier}}\right),y_{i}\right)$$

defines the expression to be minimized using the applied optimization algorithm, usually a gradient-descent-based technique. For binary classification, *L* is binary cross-entropy, where *y*_*i*_ represents the true binary label for *t*_*i*_.

It is important to point out that the embeddings obtained from BERT() are generated using the parameters $\theta_{\text{BERT}}$, which parameters are not updated during training, only $\theta_{\text{classifier}}$ are tuned.

The pretrained tokenizer is used in all cases, with application of padding to fill the maximal token length and truncation applied on exceeding it. Sparse categorical cross-entropy is applied as the loss function with Adam [62] as the optimizer. Training was terminated when the loss measured on the validation dataset stopped improving, with a patience of 10 epochs. Model performance is evaluated based on the test subset.

#### Fine-Tuning the Models

Another approach for LLM-based text classification is the fine-tuning of a pretrained model. In this case, the very same structure is applied with a classification layer at the end of the model; however, during training, the backbone model parameters are also tuned.

Let


$$\Theta \text{=}\theta_{\text{BERT}}\cup \theta_{\text{classifier}}$$

represent all parameters of the model,


$$\hat{y}\text{=}f_{\text{classifier}}\left(t,\Theta \right)$$

gives the estimated label based on input *t* and all parameters. During training


$$\underset{\Theta }{min}\frac{1}{\text{|}T\text{|}}\sum_{i\text{=}1}^{\text{|}T\text{|}}L(f_{\text{classifier}}(t_{i};\Theta ),y_{i})$$

is optimized.

During our experiments, we applied multiple learning rates to experience the effects on generalization: learning rate={10^–4^,2×10^–4^,5×10^–4^,10^–5^*,*2×10^–5^,5×10^–5^,10^–6^*,*2×10^–6^,5×10^–6^}.

To reflect model stability, each fine-tuning experiment was repeated 5 times with different random seeds. These repetitions are intended as a descriptive assessment of variability across runs; reported values represent the mean and standard deviation for these results. Given the pilot scope and limited sample size, statistical analysis was not performed in this study; more extensive testing (eg, confidence intervals and significance tests) will be included in future large-scale experiments. Tokenization and training termination are handled as described previously.

A special case in this method is the SciBERT-Longformer: as the model is untrained, a regular training is applied, with the application of the SciBERT vocabulary for tokenization.

Results are evaluated using different, well-known metrics:


$$\text{classification accuracy}\text{=}\frac{TP\text{+}TN}{P\text{+}N},$$

$$\text{precision}\text{=}\frac{TP}{TP\text{+}FP},$$

$$\text{specificity}\text{=}\frac{TN}{N},$$

$$\text{sensitivity (recall)}\text{=}\frac{TP}{P},$$

$${\text{F}}_{1}\text{-score}\text{=}\frac{2\text{*}TP}{2\text{*}TP\text{+}FP\text{+}FN},$$

where *P* and *N* refer to the number of positives and negatives, true positive (TP), true negative (TN), FP, and false negative (FN) refer to the number of correctly classified positives and negatives, and falsely classified positives and negatives, respectively.

For the screening-oriented interpretation of the best-performing fine-tuned model, additional measures were calculated from the held-out test confusion matrix. Positive predictive value (PPV) was defined as (TP/(TP+FP)), negative predictive value (NPV) as (TN/(TN+FN)), and the number of FNs was reported explicitly because missed eligible studies are critical in systematic review workflows. Workload saving was estimated as the proportion of records predicted as negative, ((TN+FN)/(P+N)), representing the share of records that would not require manual review if the classifier were used as an exclusion filter. This is reported only as an exploratory indicator; FNs would not be acceptable in an automated screening workflow. Wilson 95% CIs were calculated for proportion-based metrics to reflect uncertainty due to the small test set.

### Ethical Considerations

This study did not involve human participants, human data, or human tissue and therefore did not require approval from an institutional review board (IRB) or informed consent procedures. No identifying personal information was collected or processed, and the work adheres to relevant guidelines and regulations, including the Committee on Publication Ethics (COPE) and the principles of the Helsinki Declaration.

## Results

### Results of the Manual Review

After manual review, the number of positives is 121 (60.5%), with 79 (39.5%) negatives. The reviewers also marked those studies where checking the full text was not necessary. From the 121 positives, 22 (18.1% of positives, 11.0% of all) were marked based only on the metadata (ie, the abstract contained EQ-5D data that met our inclusion criteria), and for the remaining, the decision was made based on the full text review. For negatives, the decision was never made only on the metadata; a full study was always analyzed before the decision. Disagreements between reviewers occurred in 15 cases, but all were solved by detailed revision and discussion of the respective publications. The studies included based on abstract review (n=22) were published between 2003 and 2022, while those included based on full text review (n=99) were released between 1999 and 2022, and the excluded studies (n=79) came out between 2006 and 2022. In all the 3 manual selection subgroups, journal article (59.1%, 38.4%, and 34.2%) and research report (22.7%, 40.4%, and 32.9%) were the most frequent PubMed publication types, respectively. Although in different proportions, observational studies (9.1%, 2.0%, and 5.1%), validation studies (4.5%, 6.1%, and 5.1%), and systematic reviews (4.5%, 1%, and 5.1%) occurred in all 3 subgroups. In addition, randomized controlled trials (RCTs; n=6, 6.1%), multicenter studies (n=3, 3.0%), a review, a pragmatic clinical trial, and a book were found in the full text-based subgroup, while RCTs (n=8, 10.1%), multicenter studies (n=2, 2.5%), a review, a letter, and a twin study were found in the excluded subgroup.

### Results for BoW-Based Classification

To have a baseline architecture before applying language models, a BoW-based Naïve Bayes classifier is trained and evaluated on the same subsets, results are represented in Table 2.

**Table 2.** Precision, recall, *F*_1_-score, and accuracy for the bag-of-words-based Naïve Bayes classifier. Results clearly indicate overfitting: perfect training performance but near-random test performance. Values are weighted by class size to remove the effects of data imbalance.

	Train subset				Test subset			
Dataset	Precision	Recall	*F*_1_-score	Accuracy	Precision	Recall	*F*_1_-score	Accuracy
Title	1.00	1.00	1.00	1.00	0.53	0.53	0.53	0.53
Abstract	1.00	1.00	1.00	1.00	0.53	0.53	0.53	0.53
Title+abstract	1.00	1.00	1.00	1.00	0.53	0.53	0.53	0.53
Title+abstract+keywords	0.99	0.99	0.99	0.99	0.52	0.52	0.52	0.52

It is visible that the BoW-based approach overfitted on the training data, resulting in low performance on the test subset, which is nearly random.

### Estimation of Ground Truth Using Full-Text Analysis

The human experts labeled the documents after reviewing the document metadata and the full text, providing the ground truth for our problem in predicting the label based on only the metadata. To get an estimation of the ground truth, a full text search was performed based on the keywords defined by the human experts.

Table 3 shows the number of occurrences for each keyword and keyphrase, along with the calculated correlation coefficient. The retrieved values show a weak connection between the occurrence of each single keyword and the expected label. If a classifier is based on these values alone, a near-random classification accuracy is the result.

**Table 3.** Results of the full-text analysis: each keyword was checked in all 200 documents. Count refers to the total number of occurrences, and correlation is the correlation coefficient calculated for each keyword and the actual label.

Keyword	Count	Pearson correlation coefficient
Index	150	0.24
Value	182	0.25
Utility	101	0.08
Utilities	47	0.13
Score	187	0.16
Mobility	136	0.19
Self-care	106	0.22
Usual activities	91	0.20
Pain	181	0.16
Discomfort	119	0.23
Anxiety	139	0.20
Depression	143	0.19
Looking after myself	2	0.08
Doing usual activities	2	0.08
Having pain or discomfort	2	0.08
Feeling worried	1	0.06
Feeling sad	0	—^a^
Feeling unhappy	0	—

a Not applicable.

**Figure 3.**
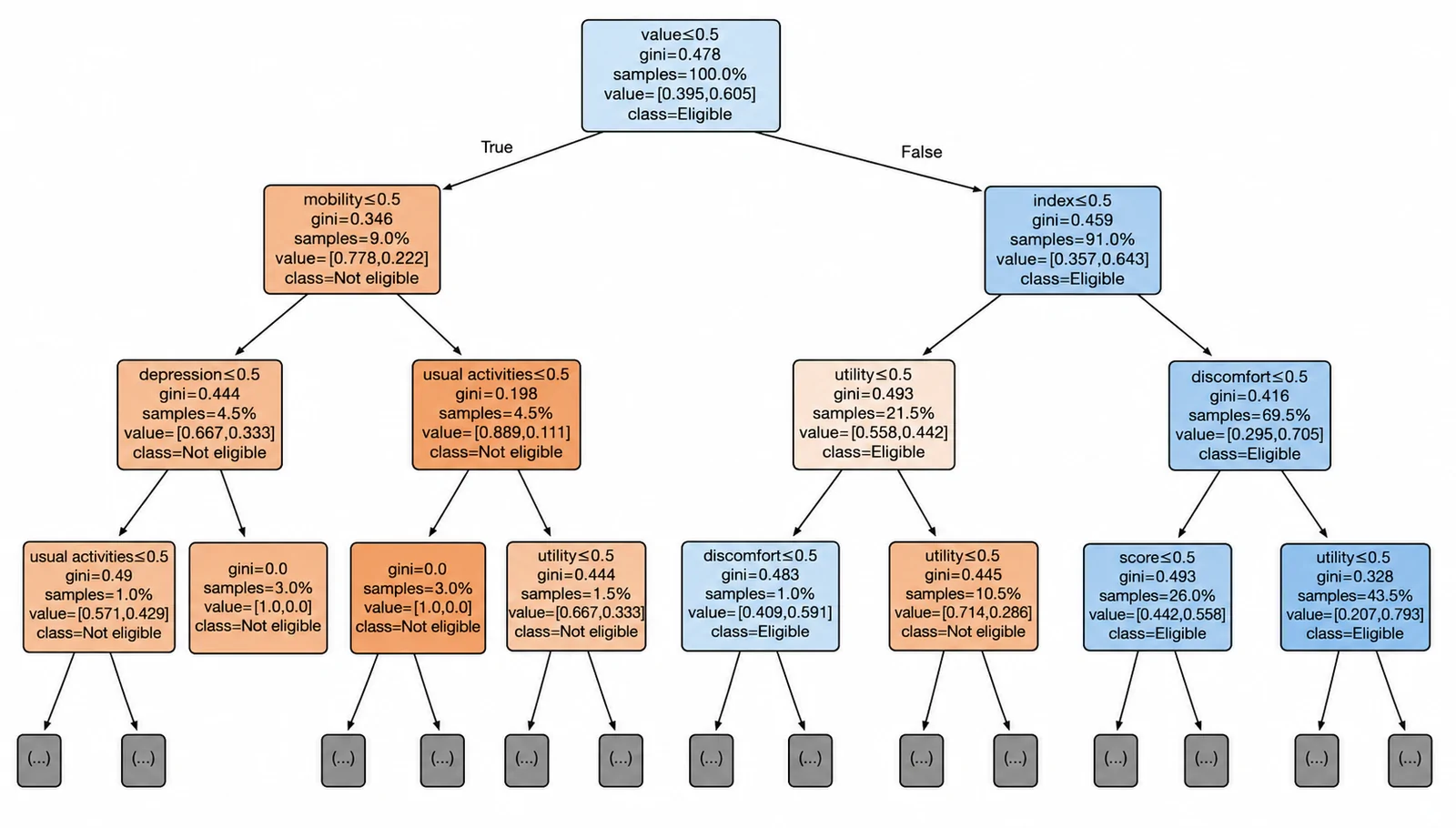
Visualization of the decision tree trained on the analyzed keywords of the full-text data. For readability, only the first 3 levels of the tree are shown. Each internal node represents a decision, while leaf nodes represent the outcomes. The first line in each node shows the expression as “k \leq 0.5” for a given keyphrase ‘k,’ which can be interpreted as “if the document does not contain keyphrase ‘k.’” The left subtree represents nodes where the expression is evaluated as true; the right subtree represents nodes where the evaluation is false. Other labels in the nodes include the gini impurity value, sample shows the proportion of all elements affected at a given node, and values show the mean of each value for each class.

The trained DT is visualized in Figure 3. The depth of the full tree is 12 levels, and the classification accuracy is 87%. It is worth mentioning that DTs with a limited depth also give similar performance: an 8-level DT scores 82.5%, and a DT with 6 levels results in a 78.5% classification accuracy. The goal of this analysis was to find the relationship between the target label and keywords and their different combinations; therefore, no test subset was selected, meaning that the results are fully fitted to the whole dataset.

The results of this experiment show that the analysis of the full text is complicated, while the application of high-performing document-based classification methods is not possible without document, journal, or publisher-based preprocessing; a basic dictionary-based method fails to close on the performance of the domain experts.

### Comparing Different Pretrained Language Models

The following experiment consisted of applying the pretrained language model without altering the parameters, only training the classification layer. Results are detailed in Table 4. It is easy to conclude that while results in some cases are above the near-random BoW baseline, performance falls short of the generally accepted marks.

**Table 4.** Performance of pretrained Bidirectional Encoder Representations from Transformers (BERT)–family models without alterations; only the classifier parameters were trained. Reported metrics of the table include accuracy, precision, recall, and *F*_1_-score for the highest performed configuration of each model and dataset (defined by top test accuracy).

Model and applied dataset		Accuracy	Precision	Recall	*F*_1_-score
BERT^a^					
Title		0.63	0.66	0.63	0.54
Abstract		0.68	0.73	0.68	0.62
Title + abstract		0.66	0.71	0.66	0.59
Title + abstract+keywords		0.61	0.76	0.61	0.47
BioBERT^b^					
Title		0.65	0.66	0.65	0.59
Abstract		0.67	0.68	0.67	0.63
Title +abstract		0.66	0.65	0.66	0.63
Title + abstract+keywords		0.62	0.6	0.62	0.59
SciBERT^c^					
Title		0.67	0.68	0.67	0.63
Abstract		0.64	0.78	0.64	0.53
Title+abstract		0.64	0.66	0.64	0.57
Title+abstract+keywords		0.64	0.66	0.64	0.57
BlueBERT^d^					
Title		0.64	0.78	0.64	0.53
Abstract		0.63	0.77	0.63	0.51
Title+abstract		0.66	0.71	0.66	0.59
Title+abstract+keywords		0.62	0.77	0.62	0.49

a BERT: Bidirectional Encoder Representations from Transformers.

b BioBERT: Biomedical Bidirectional Encoder Representations from Transformers.

c SciBERT: Scientific Bidirectional Encoder Representations from Transformers.

d BlueBERT: Biomedical Language Understanding Evaluation Bidirectional Encoder Representations from Transformers.

Experiments were done in a multiple central processing units environment, while graphics processing unit acceleration is also possible. Popular frameworks (*TensorFlow*, *transformers*, and *sklearn*) were applied in implementation with generic hyperparameters; details are available in the source codes that are made publicly available.

It is also trivial that when only the title or only the abstract is submitted, it is outperformed by solutions with more input data.

### Fine-Tuning the Language Models

When applying the LLMs in a transfer learning scenario, where pretrained values are used as initial parameters and are allowed to change during training, it is empirically determined that performance is higher.

In these experiments, we used the dataset where the title, the abstract, and the keywords are concatenated into a single sequence.

Results show (Table 5) that the top-performing model was the BioBERT on a specific learning rate, which slightly outperformed the BERT and the Longformer models.

**Table 5.** The results of fine-tuning different large language models (LLMs) with varying learning rates, sorted by classification accuracy, in descending order. All trainings were done 5 times, average accuracy, and the SD is also presented next to precision, recall, and *F*_1_-score measured on the test subset.

Model	Learning rate	Accuracy	Accuracy, mean (SD)	Precision	Recall	*F*_1_-score
BERT^a^	0.000002	0.70	0.67 (0.02)	0.71	0.70	0.68
BioBERT^b^	0.000050	0.70	0.66 (0.02)	0.71	0.70	0.68
BERT	0.000001	0.69	0.67 (0.02)	0.74	0.69	0.64
BERT	0.000100	0.69	0.64 (0.03)	0.69	0.69	0.69
BERT	0.000010	0.69	0.67 (0.01)	0.69	0.69	0.67
BlueBERT^c^	0.000100	0.69	0.60 (0.07)	0.69	0.69	0.69
SciBERT^d^ Long	0.000100	0.69	0.66 (0.02)	0.76	0.69	0.63
BioBERT	0.000005	0.68	0.65 (0.03)	0.68	0.68	0.66
SciBERT	0.000100	0.68	0.64 (0.02)	0.70	0.68	0.64
BlueBERT	0.000005	0.68	0.62 (0.04)	0.67	0.68	0.67

a BERT: Bidirectional Encoder Representations from Transformers.

b BioBERT: Biomedical Bidirectional Encoder Representations from Transformers.

c BlueBERT: Biomedical Language Understanding Evaluation Bidirectional Encoder Representations from Transformers.

d SciBERT: Scientific Bidirectional Encoder Representations from Transformers.

The screening-oriented metrics (Table 6) show that the best-performing fine-tuned configuration achieved high sensitivity but low specificity. This indicates that the model was able to identify most eligible EQ-5D data reports, but at the cost of a substantial number of FPs. The false-negative count remained nonnegligible; therefore, the model should not be interpreted as a safe autonomous exclusion tool in its current form; instead, these results support early technical feasibility.

**Table 6.** Screening-oriented performance of the best-performing fine-tuned Bidirectional Encoder Representations from Transformers (BERT) configuration on the held-out test set. Wilson 95% CIs are reported for proportion-based metrics. The confusion matrix was true positive (TP)=54, true negative (TN)=16, false positive (FP)=24, and false negative (FN)=6.

Metric	Value	Wilson 95% CI
Accuracy	70.0%	60.4%‐78.1%
Sensitivity	90.0%	79.9%‐95.3%
Specificity	40.0%	26.3%‐55.4%
PPV^a^	69.2%	58.3%‐78.4%
NPV^b^	72.7%	51.8%‐86.8%
False negatives	6	—^c^
*F*_1_-score	0.783	—

a PPV: positive predictive value.

b NPV: negative predictive value.

c Not applicable.

A supplementary 10-run stability analysis of the same learning-rate configuration showed test accuracy ranging from 62.0% to 70.0% (mean 66.3%, SD 2.4) and FNs ranging from 2 to 11 across seeds.

The test set contains 7 positive records where the reviewers were able to decide based only on the metadata; the model classified all of them correctly. It is worth mentioning that the training of the SciBERT longformer does not classify as fine-tuning, as the model parameters were trained from scratch. It is also visible that the BlueBERT and SciBERT models were only slightly outperformed; however, learning rate selection has a significant effect on the outcome.

It is worth noting that several model configurations achieved similar performance; however, small differences in accuracy between the top models should be interpreted cautiously. The observed performance may change under alternative train/validation/test splits or under additional training repetitions. Therefore, this reported ordering of high-performing models is presented primarily as preliminary feasibility evidence.

To ensure comparability across models, we report accuracy, precision, recall, specificity, and *F*_1_-score consistently in all experiments. Future work will also include statistical analysis (eg, CIs and significance testing) to better assess model differences.

## Discussion

### Principal Findings

We analyzed LLM-based methods to select EQ-5D data reports (either the EQ-5D index score or results of the EQ-5D descriptive system) in manuscripts in PubMed based on the publicly available metadata such as the title, abstract, and keywords. In the experiments, we tested the performance of multiple domain-specific pretrained models, with different architectures and setups. We found that top performance is achieved by applying fine-tuning to the models. We also found that estimating the ground truth from the full text has limitations, further emphasizing the importance of study metadata-based methods.

According to our best knowledge, this is the first study aiming to identify studies reporting EQ-5D data using the LLM-based automation method. Therefore, direct comparisons with the international literature are not feasible. Tóth et al [3] in their recent review have identified in PubMed 108 studies on SLR automation methods and a further 15 SLRs that applied automation partly or throughout the SLR process. Automation of record screening (based on title and abstract) was the most frequently studied SLR stage, and, similarly to our approach, typically manually screened publications were used for the training of the ML classifier. The BERT model and other LMs were applied in recent studies, presenting the increasing use of novel NLP techniques. Torre-Lopez et al [63] also reported that interest is clearly increasing in the field of SLR automation. Sundaram and Berleant [64] highlighted that there are gaps in the application of different text mining and NLP methods in different areas of the automated SLR process. Hasny et al [65] applied different BERT models for paper screening and reported a significant reduction in necessary human workload.

For the interpretation of our results, some limitations of our study have to be noted. The most significant limitation is definitely the small sample of publications that we used to train and test our model. Nonetheless, this first experimental study aimed to identify methodological challenges, such as feasibility, and formulate some points to consider for which a smaller study was deemed sufficient. Our future plans include the extension of the data using semisupervised methods based on the current models, which will hopefully lower the time cost per study.

Another related limitation is that the results are based on a single, predefined split of the small dataset; therefore, estimates are split-dependent and do not support strong generalization claims. Repeated cross-validation and/or external validation on independent samples is required to assess reliability and stability. Although sensitivity was relatively high in the best-performing configuration, specificity remained limited, and FNs were still observed. Therefore, the model is not suitable for unsupervised exclusion of records in systematic review workflows.

We would like to highlight some aspects of our study that we think are worthwhile for future research in the field.

First, we did not restrict our PubMed search to specific publication or study types but considered all hits (eg, books, reviews, and twin studies, etc). Given that different types of publications and studies have different reporting designs and standards, we assume that our model would have yielded different (presumably more precise) results if we had investigated automation, for instance, only among RCTs. RCTs are one of the most frequently searched publications for health technology assessment, as RCTs provide the highest level of clinical evidence. Reporting standards for RCTs have been established and are being updated [66], thus can be used as a practical gold standard for the authors on how to write the studies and to consider these reporting requirements already at the designing phase of the trials. With regard to outcomes, CONSORT (Consolidated Standards of Reporting Trials) requires to report “Completely defined prespecified primary and secondary outcome measures, including how and when they were assessed” (Methods section), and “For each primary and secondary outcome, results for each group, and the estimated effect size and its precision (such as 95% CI)” (Results section). Moreover, the CONSORT extension [67] for abstracts points out that primary outcomes and their results should be reported in the abstract of the study. Therefore, we think it would be an interesting avenue for further research to test our automation method on specific study types, and RCTs could be one of the firsts, given their importance and the relatively high-quality publication practice.

Second, many publications (eg, some RCTs but more probably the observational studies, real-world data reports, etc) still do not follow the reporting standards and do not describe all important details in a complete and transparent way. Although the development of LLMs alone may bring some increase in the precision of literature review automation, we believe that no breakthrough can be achieved without improving broadly the reporting practice. This latter is a huge responsibility of authors and probably even more so of journal editors.

Third, we also believe that developers of reporting guidelines should pay more attention to the high need for automation of systematic reviews and consider the capabilities and needs of AI-based automated search, selection, and data extraction technologies. For instance, text mining and data extraction from tables and figures are much more challenging, or even not feasible for LLMs when compared to processing plain text. According to our best knowledge, this aspect has not been considered in reporting guidelines up to now. The EQUATOR (Enhancing the Quality and Transparency of health Research) Network [68] gives recommendations for reporting guideline development and emphasizes the importance of inviting stakeholders with a wide range of expertise in the development group. These usually include journal editors, statisticians, epidemiologists, clinicians, but the potential role of IT experts in natural language processing has not come into focus so far.

Fourth, the EQ-5D outcome measurement tool was in the center of our study and the publication pool (out of which we randomly selected our study sample of 200 records) involved all pieces since its inception in 1990. The EQ-5D is somewhat an exceptional outcome measurement tool compared to others due to its more than 3 decades of history and, perhaps more importantly, being backed by a professional team of researchers, the EuroQol Group. Significant progress around the EQ-5D use and data sharing has been seen in the past years. For instance, the EQ-5D terminology has been established (and is being maintained), a PubMed search strategy has been developed, recommendations on how to analyze and report EQ-5D data have been published, and educational materials and user guides have been launched in various languages. Hence, the generalizability of our findings to reviews of other outcome measures with a more modest literature background and reporting standards requires further investigation. The exclusion of EQ VAS-only studies should also be considered when interpreting the results: this choice reflects the operational target of this study, namely the identification of publications reporting EQ-5D descriptive-system or index-score data. Nonetheless, it would be worth investigating (on a larger sample of EQ-5D publications) whether the more recent studies (eg, from the past 10 years) are in fact more “readable” for LLMs than the older ones. Fifth, we find it important to highlight that our methods and results may not be directly applicable to searches in other databases. Literature databases (eg, Embase and Scopus) may differ in many respects, that is, the list of journals indexed and the types of publications recorded, and also how they handle metadata for non-English language publications. All these may have implications on the performance of the LLMs in selecting publications. We encourage researchers to adapt and put our approach to the test in other biomedical literature databases.

### Conclusion

This study demonstrates the feasibility of automating the identification of EQ-5D data reports in PubMed based on scientific publication metadata using LLMs. Although accuracy remains moderate due to the limited dataset size, the approach successfully reproduces manual screening trends and establishes a reproducible framework for future large-scale and semisupervised analyses. It is important to point out that the current model is not applicable for autonomous screening or unsupervised exclusion of records; this study was designed as a proof-of-concept evaluation of whether EQ-5D data reporting in the full text of biomedical publications can be predicted from PubMed metadata alone.

As the results of this study show moderate performance, it itself is not applicable for autonomous screening; however, it provides a foundation for future studies using larger datasets.

To efficiently increase the training data, we aim to design a semisupervised approach, where reviewers are supported by the estimations from our model, along with a confidence value. We believe that by sampling the available data considering the estimations and certainty, a process of reviewing could be designed, where time-consuming is minimized.

The current model is not ready for automated screening or unsupervised exclusion of records. Its potential use is to support human reviewers by prioritizing records or flagging likely candidates for further assessment.

With an increased number of training samples, different models might outperform the currently used fine-tuned BioBERT; pretrained models or an untrained architecture (eg, the SciBERT Longformer) could achieve a higher performance. Other architectures, multiheaded BERT structures, or ensemble classifiers based on multiple trained models could also be used to improve performance. Robustness and generalizability require validation using external datasets in future work.

## Abbreviations

BERT: Bidirectional Encoder Representations from Transformers
biLM: bidirectional language model
BioBERT: Biomedical Bidirectional Encoder Representations from Transformers
BlueBERT: Biomedical Language Understanding Evaluation Bidirectional Encoder Representations from Transformers
BoW: bag-of-words
CONSORT: Consolidated Standards of Reporting Trials
COPE: Committee on Publication Ethics
DT: decision tree
EQ VAS: EuroQol Visual Analogue Scale
EQUATOR: Enhancing the Quality and Transparency of health Research
FN: false negative
FP: false positive
HRQoL: health-related quality of life
IRB: institutional review board
LLM: large language model
ML: machine learning
NLP: natural language processing
NPV: negative predictive value
PPV: positive predictive value
PROM: patient-reported outcome measure
QALY: quality-adjusted life year
RCT: randomized controlled trial
SciBERT: Scientific Bidirectional Encoder Representations from Transformers
SLR: systematic literature review
TF-IDF: Term Frequency-Inverse Document Frequency
TN: true negative
TP: true positive
VAS: visual analog scale
WSS@95: workload savings at 95% recall

## References

[1] Cochrane handbook for systematic reviews of interventions (current version). Cochran.

[2] Zah V, Burrell A, Asche C, Zrubka Z (2022). Paying for digital health interventions – what evidence is needed?. Acta Polytech Hung.

[3] Tóth B, Berek L, Gulácsi L, Péntek M, Zrubka Z (2024). Automation of systematic reviews of biomedical literature: a scoping review of studies indexed in PubMed. Syst Rev.

[4] Blaizot A, Veettil SK, Saidoung P (2022). Using artificial intelligence methods for systematic review in health sciences: a systematic review. Res Synth Methods.

[5] Kowsari K, Jafari Meimandi K, Heidarysafa M, Mendu S, Barnes L, Brown D (2019). Text classification algorithms: a survey. Information.

[6] LeCun Y, Bengio Y, Hinton G (2015). Deep learning. Nature.

[7] Brants T, Popat A, Xu P, Och FJ, Dean J Large language models in machine translation. https://aclanthology.org/volumes/D07-1/.

[8] Floridi L, Chiriatti M (2020). GPT-3: its nature, scope, limits, and consequences. Minds Mach.

[9] Min B, Ross H, Sulem E (2024). Recent advances in natural language processing via large pre-trained language models: a survey. ACM Comput Surv.

[10] HaCohen-Kerner Y, Miller D, Yigal Y (2020). The influence of preprocessing on text classification using a bag-of-words representation. PLoS One.

[11] Selva Birunda S, Kanniga Devi R A review on word embedding techniques for text classification.

[12] Peters M, Neumann M, Zettlemoyer L, Yih W tau Dissecting contextual word embeddings: architecture and representation. http://aclweb.org/anthology/D18-1.

[13] Devlin J, Chang MW, Lee K, Toutanova K (2018). Bert: pre-training of deep bidirectional transformers for language understanding. arXiv.

[14] Radford A, Wu J, Child R, Luan D, Amodei D, Sutskever I (2019). Language models are unsupervised multitask learners. https://cdn.openai.com/better-language-models/language_models_are_unsupervised_multitask_learners.pdf.

[15] Vaswani A, Shazeer N, Parmar N, Uszkoreit J, Jones L, Gomez AN (2017). Advances in Neural Information Processing Systems 30.

[16] Meadows KA (2011). Patient-reported outcome measures: an overview. Br J Community Nurs.

[17] Search for EQ-5D documents. EuroQol.

[18] Devlin NJ, Brooks R (2017). EQ-5D and the EuroQol group: past, present and future. Appl Health Econ Health Policy.

[19] Longworth L, Yang Y, Young T (2014). Use of generic and condition-specific measures of health-related quality of life in NICE decision-making: a systematic review, statistical modelling and survey. Health Technol Assess.

[20] White J (2020). PubMed 2.0. Med Ref Serv Q.

[21] González-Carvajal S, Garrido-Merchán EC (2021). Comparing BERT against traditional machine learning text classification. arXiv.

[22] Gasparetto A, Marcuzzo M, Zangari A, Albarelli A (2022). A survey on text classification algorithms: from text to predictions. Information.

[23] Dogra V, Verma S (2022). A complete process of text classification system using state-of-the-art NLP models. Comput Intell Neurosci.

[24] Li Q, Peng H, Li J (2022). A survey on text classification: from traditional to deep learning. ACM Trans Intell Syst Technol.

[25] Zhou C, Li Q, Li C (2025). A comprehensive survey on pretrained foundation models: a history from BERT to ChatGPT. Int J Mach Learn Cybern.

[26] Alammary AS (2022). BERT models for Arabic text classification: a systematic review. Applied Sciences.

[27] Almaliki M, Almars AM, Gad I, Atlam ES (2023). ABMM: Arabic BERT-mini model for hate-speech detection on social media. Electronics (Basel).

[28] Li J, Lin Y, Zhao P (2022). Automatic text classification of actionable radiology reports of tinnitus patients using bidirectional encoder representations from transformer (BERT) and in-domain pre-training (IDPT). BMC Med Inform Decis Mak.

[29] Osváth M, Yang ZG, Kósa K (2023). Analyzing narratives of patient experiences: a BERT topic modeling approach. Acta Polytech Hung.

[30] Lee JS, Hsiang J (2020). Patent classification by fine-tuning BERT language model. World Pat Inf.

[31] Sun C, Qiu X, Xu Y, Huang X How to fine-tune bert for text classification? chinese computational linguistics.

[32] Adhikari A, Ram A, Tang R, Lin J (2019). DocBERT: BERT for document classification. arXiv.

[33] Zheng S, Yang M A new method of improving bert for text classification.

[34] Zhang T, Kishore V, Wu F, Weinberger KQ, Artzi Y (2020). Bertscore: evaluating text generation with bert. arXiv.

[35] Reimers N, Gurevych I (2019). Sentence-BERT: sentence embeddings using siamese BERT-networks. https://www.aclweb.org/anthology/D19-1.

[36] Khadhraoui M, Bellaaj H, Ammar MB, Hamam H, Jmaiel M (2022). Survey of BERT-base models for scientific text classification: COVID-19 case study. Applied Sciences.

[37] Beltagy I, Lo K, Cohan A SciBERT: a pretrained language model for scientific text. https://www.aclweb.org/anthology/D19-1.

[38] Lee J, Yoon W, Kim S (2020). BioBERT: a pre-trained biomedical language representation model for biomedical text mining. Bioinformatics.

[39] Peng Y, Yan S, Lu Z Transfer learning in biomedical natural language processing: an evaluation of BERT and elmo on ten benchmarking datasets.

[40] Beltagy I, Peters ME, Cohan A (2020). Longformer: the long-document transformer. arXiv.

[41] Mutasodirin MA, Prasojo RE (2021). 2021 International Conference on Advanced Computer Science and Information Systems (ICACSIS.

[42] Yu S, Su J, Luo D (2019). Improving BERT-based text classification with auxiliary sentence and domain knowledge. IEEE Access.

[43] Wang B, Xie Q, Pei J (2024). Pre-trained language models in biomedical domain: a systematic survey. ACM Comput Surv.

[44] Mosbach M, Andriushchenko M, Klakow D On the stability of fine-tuning BERT: misconceptions, explanations, and strong baselines. https://openreview.net/forum?id=nzpLWnVAyah.

[45] Gates A, Gates M, Sebastianski M, Guitard S, Elliott SA, Hartling L (2020). The semi-automation of title and abstract screening: a retrospective exploration of ways to leverage Abstrackr’s relevance predictions in systematic and rapid reviews. BMC Med Res Methodol.

[46] Hamel C, Kelly SE, Thavorn K, Rice DB, Wells GA, Hutton B (2020). An evaluation of DistillerSR’s machine learning-based prioritization tool for title/abstract screening - impact on reviewer-relevant outcomes. BMC Med Res Methodol.

[47] Ferdinands G, Schram R, de Bruin J (2023). Performance of active learning models for screening prioritization in systematic reviews: a simulation study into the average time to discover relevant records. Syst Rev.

[48] Gilardi F, Alizadeh M, Kubli M (2023). ChatGPT outperforms crowd workers for text-annotation tasks. Proc Natl Acad Sci U S A.

[49] Loukas L, Stogiannidis I, Malakasiotis P, Vassos S (2023). Breaking the bank with ChatGPT: few-shot text classification for finance. arXiv.

[50] Guo E, Gupta M, Deng J, Park YJ, Paget M, Naugler C (2024). Automated paper screening for clinical reviews using large language models: data analysis study. J Med Internet Res.

[51] Dennstädt F, Zink J, Putora PM, Hastings J, Cihoric N (2024). Title and abstract screening for literature reviews using large language models: an exploratory study in the biomedical domain. Syst Rev.

[52] Tran VT, Gartlehner G, Yaacoub S (2024). Sensitivity and specificity of using GPT-3.5 Turbo models for title and abstract screening in systematic reviews and meta-analyses. Ann Intern Med.

[53] Matsui K, Utsumi T, Aoki Y, Maruki T, Takeshima M, Takaesu Y (2024). Human-comparable sensitivity of large language models in identifying eligible studies through title and abstract screening: 3-layer strategy using GPT-3.5 and GPT-4 for systematic reviews. J Med Internet Res.

[54] Li M, Sun J, Tan X (2024). Evaluating the effectiveness of large language models in abstract screening: a comparative analysis. Syst Rev.

[55] Chai KEK, Lines RLJ, Gucciardi DF, Ng L (2021). Research screener: a machine learning tool to semi-automate abstract screening for systematic reviews. Syst Rev.

[56] Papaioannou D, Brazier J, Paisley S (2013). Systematic searching and selection of health state utility values from the literature. Value Health.

[57] Arber M, Garcia S, Veale T, Edwards M, Shaw A, Glanville JM (2017). Performance of ovid medline search filters to identify health state utility studies. Int J Technol Assess Health Care.

[58] Group TE (1990). EuroQol - a new facility for the measurement of health-related quality of life. Health Policy.

[59] Rencz F, Brodszky V, Gulácsi L (2020). Parallel valuation of the EQ-5D-3L and EQ-5D-5L by time trade-off in Hungary. Value Health.

[60] Herdman M, Gudex C, Lloyd A (2011). Development and preliminary testing of the new five-level version of EQ-5D (EQ-5D-5L). Qual Life Res.

[61] Golicki D, Młyńczak K (2022). Measurement properties of the EQ-5D-Y: a systematic review. Value Health.

[62] Kingma DP, Ba J (2017). Adam: a method for stochastic optimization. arXiv.

[63] de la Torre-López J, Ramírez A, Romero JR (2023). Artificial intelligence to automate the systematic review of scientific literature. Computing.

[64] Sundaram G, Berleant D (2023). International Congress on Information and Communication Technology.

[65] Hasny M, Vasile AP, Gianni M, Bannach-Brown A, Nasser M, Mackay M BERT for complex systematic review screening to support the future of medical research.

[66] Moher D, Hopewell S, Schulz KF (2010). CONSORT 2010 explanation and elaboration: updated guidelines for reporting parallel group randomised trials. BMJ.

[67] Hopewell S, Clarke M, Moher D (2008). CONSORT for reporting randomised trials in journal and conference abstracts. The Lancet.

[68] Simera I, Moher D, Hirst A, Hoey J, Schulz KF, Altman DG (2010). Transparent and accurate reporting increases reliability, utility, and impact of your research: reporting guidelines and the EQUATOR Network. BMC Med.

[69] Towards automating the selection of articles reporting EQ-5D data for systematic literature reviews using large language models. GitHub.

